# Monthly Nursing

**Published:** 1890-04

**Authors:** 


**Monthly Nursing.** By A. WORCESTER, A. M., M. D., Fellow af the Massachusetts Medical Society; Physician to the Waltham Hospital. Second Edition. 12 mo, p. 250. New York: D. Appleton & Company. 1890.

When the first edition of this book appeared a few years ago, it was well received, and we are of the opinion that it merited the favor of the profession, and the nurses for whom it was more especially written. The author has had a large experience in obstetric practice, and in the training of nurses for this special field. That a second edition has become necessary, speaks in support of its value, even more than any journalistic endorsement. It is full of good teaching for the obstetric nurse, and may well repay perusal by physicians. It is one of the first books of its kind that we have seen, and we advise all nurses to study it with care. In an appendix will be found much of importance, and it is a well-printed, handsome volume.

